# Small particles of *Echinococcus granulosus* (spegs) and *Echinococcus multilocularis* (spems) promote follicular T helper cell expansion and are associated with IgE and IgG4 class switching in human lymph nodes

**DOI:** 10.1186/s13071-026-07321-4

**Published:** 2026-03-18

**Authors:** Leonard Schreiber, Linyun Zhang, Johannes Grimm, Kevin Mellert, Juliane Nell, Peter Möller, Thomas F. E. Barth

**Affiliations:** Institute of Pathology of the University Ulm, Ulm, Germany

**Keywords:** Echinococcosis, Follicular helper T cells, Immunomodulation, CD57, IgG4, IgE

## Abstract

**Background:**

Cystic echinococcosis (CE) and alveolar echinococcosis (AE) are zoonotic diseases caused by the larval stages of *Echinococcus granulosus* and *E. multilocularis*. Prior studies have identified small particles from the outer layer of parasitic lesions termed spegs (from *E. granulosus*) and spems (from *E. multilocularis*), which appear to interact with the human immune system, leading to lymph node enlargement.

**Methods:**

We analyzed lymph nodes from CE (*n* = 3) and AE (*n* = 3) patients by immunohistochemical staining for spegs/spems and CD57. Automated image analysis revealed a significantly increased number of CD57-positive cells in *Echinococcus* particle-positive germinal centers compared with speg-/spem-negative germinal centers.

Double immunofluorescence staining was used to characterize CD57-positive cells with markers for CD2, CD3, CD4, CD8, PD-1, Granzyme B, Perforin, and T-cell intracellular antigen 1 (TIA1). Additionally, IgG4 and IgE immunostaining was performed on liver lesions and lymph nodes containing spegs and spems and compared with unaffected controls.

**Results:**

Spegs and spems were found to accumulate in the germinal centers of the lymph nodes. Affected germinal centers showed a higher number of CD57-positive cells, which coexpressed CD2, CD3, CD4, and PD-1 but lacked cytotoxic markers (CD8, Granzyme B, Perforin, TIA1), identifying them as follicular helper T cells (Tfh). Furthermore, the speg- and spem-affected liver and lymph node sections exhibited significantly increased numbers of IgG4-, IgE-, and CD57-positive cells.

**Conclusions:**

Spegs and spems are associated with changes in the host immune response, characterized by an increased accumulation of Tfh in germinal centers. This finding is accompanied by the presence of IgE and IgG4 plasma cells in the lymph node and the perilesional area, suggesting parasite-associated immunomodulatory processes in echinococcosis.

**Graphical Abstract:**

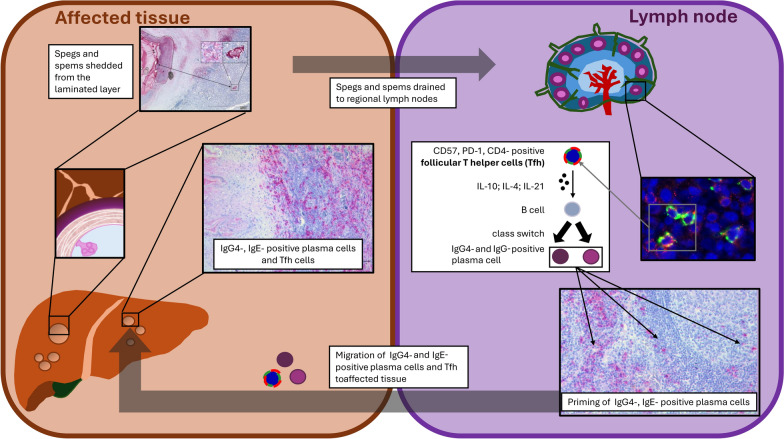

**Supplementary Information:**

The online version contains supplementary material available at 10.1186/s13071-026-07321-4.

## Background

Echinococcosis is a zoonosis caused by *Echinococcus granulosus* and *E. multilocularis*, leading to cystic and alveolar echinococcosis in humans. These infections result in pseudocystic lesions consisting of an inner germinal layer and an outer laminated layer (LL), mainly composed of glycoproteins.

Small particles shed from the LL have previously been referred to as small particles of *E. granulosus* (spegs) and *E. multilocularis* (spems). These particles are detectable in lymph nodes draining the infected tissue, but their immunological role remains unclear.

Tfh are implicated in several parasitic diseases. IgG4- and IgE-producing plasma cells are known to be increased in response to parasite infections.

In this study, we examined human lymph nodes and liver lesions from CE and AE patients containing spegs or spems. We observed an accumulation of CD57-positive cells in the germinal centers of the lymph nodes, which were further identified immunohistochemically as Tfh by co-expression of CD2, CD3, CD4, and PD-1 and the absence of cytotoxic markers. Additionally, IgG4- and IgE-positive plasma cells were found at increased levels in lymph node and liver sections.

These findings suggest that spegs and spems contribute to a parasite-specific immune modulation, characterized by an enhanced Tfh response and increased IgG4- and IgE-associated plasma cell presence in echinococcosis.

The larval stages of cestode parasites belonging to the *Echinococcus granulosus* and *Echinococcus multilocularis* species complex cause cystic and alveolar echinococcosis in humans [1, 2]. These two diseases have particular clinical relevance, as they have a wide geographic distribution and may cause life-threatening disease [2]. During the larval stage, the metacestodes of these parasites form pseudocysts, primarily in the human liver. These pseudocysts consist of an inner germinal cell layer and an outer acellular layer, called the laminated layer (LL). The LL is mainly composed of a mucin meshwork containing O-linked glycans with a high concentration of galactose [3]. The cystic structures persist and progressively grow in the infected organs over many years, leading to destruction of the surrounding liver tissue [4]. During cyst growth, particles from the LL are shed and drained to the lymph nodes. We introduced the terms small particles of *Echinococcus granulosus* (spegs) and *Echinococcus multilocularis* (spems) to describe these particles [5–7]. We further identified these small particles from the LL in the surrounding tissue and in germinal centers of draining enlarged lymph nodes in humans [8]. As these particles accumulate in the germinal centers of affected lymph nodes, their interactions with the immune system are of particular interest and may provide important insights into host-parasite immune interplay [9].

The LL, the source of the particles, has various immune-modulating functions. The acellular glycoprotein-rich layer protects the germinal layer from leukocyte invasion and allows the diffusion of macromolecules, impacting parasite survival [10]. The LL is detected by immunohistochemical staining (IHC) with the monoclonal antibody (mAb) EmG3 in tissue infected by the larval stage of *Echinococcus*. Using these antibodies, we detected these particles, which are shed into the surrounding tissue and lymph nodes during cyst growth [7, 11]. Since these particles do not contain DNA [12], they can be considered non-infectious. Nevertheless, the enlargement of spem- and speg-positive lymph nodes indicates an interaction of these particles with immune cells [8]. Whether this interaction contributes to disease progression or host defense and, if so, which immune cell populations are involved remain unclear.

CD57-positive T cells have emerged as key mediators of immune regulation, particularly in settings of chronic antigen exposure and parasitic infection [13]. Rather than functioning merely as terminally differentiated or exhausted lymphocytes, CD57-expressing T cells, including Tfh, represent a distinct subset with specialized immunomodulatory capabilities. These cells are characterized by robust effector functions, enhanced cytokine production, and the capacity to shape local immune responses [14–16]. Their expression of regulatory molecules such as PD-1 further underscores their active involvement in fine-tuning germinal center dynamics and coordinating adaptive immunity rather than simply reflecting cellular senescence [17]. Accumulating evidence indicates that CD57-positive T cells play a central role in orchestrating immune responses during chronic infections, influencing disease outcome and potentially contributing to pathogen-driven immune modulation [15, 18, 19].

In this study, we aimed to characterize the involvement of spegs and spems in the immune response. To this end, we analyzed human tissue samples using computer-based immunohistochemistry and double-immunofluorescence staining for spegs, spems, and CD57-positive cells. We further examined lymph nodes and liver tissue for IgG4- and IgE-positive plasma cells, as these antibody-producing cells are known to be involved in the immune response to *Echinococcus* infection [20].

## Methods

The antibodies used in this study are listed in Table S1 in the supplementary material.

First, a series of six lymph nodes from humans with *E. granulosus* (*n* = 3) and *E. multilocularis* (*n* = 3) was stained using a standard immunohistochemical (IHC) protocol with the monoclonal antibodies EmG3 and a CD57, as described elsewhere [8, 21]. Previous studies have shown that our method is suitable for tissue samples > 20 years old [7]. These draining lymph nodes stem from hepatic (*n* = 4) and pulmonary (*n* = 2) lesions. From these blocks, serial tissue sections were obtained. Detailed patient characteristics are listed in Table S2 in the supplementary material.

The stained tissue sections were scanned, and the images of the two stained antibodies were aligned with their corresponding regions using the Slidematch^™^ software by microDimensions GmbH. For further image analysis, CellProfiler^™^ 4.2.4 software by Broad Institute [22] was used. The software identified spegs, spems, and CD57-positive germinal centers. Additionally, pictures of EmG3-negative germinal centers and their CD57-stained germinal centers were taken by a charge-coupled device (CCD) camera coupled to a computer using CellProfiler^™^. The number of CD57-positive cells in the germinal centers was identified and compared with the intensity of the corresponding germinal center in the EmG3 staining. The intensity of the defined region was quantified using CellProfiler^™^, which calculated the mean pixel intensity within the red channel. To ensure staining intensity comparability across sections, the EmG3 intensity values were normalized to the maximum signal measured in each lymph node. Moreover, the ratio of CD57-positive cells to the total cell counts in EmG3-positive and -negative germinal centers was calculated. The total cell count was obtained by dividing the specified area by the average cell size measured.

Statistical analysis of the number of CD57-positive cells and EmG3 intensity was performed using Spearman’s rank correlation coefficient. Statistical significance was assessed using the *t* approximation for Spearman’s correlation.

Four different lymph nodes with germinal centers positive for spegs and spems in immunohistochemical staining were further analyzed by double immunofluorescence. Patient details are given in Table S3 in the supplementary material. As control, we used three lymph nodes and one tonsil of non-infected humans. The exact procedure was described by Barrios et al. [5].

For immunofluorescent staining, we used CD57 antibody conjugated with Alexa Fluor 488 (green); the corresponding antibodies to detect further markers (CD2, CD3, CD 4, CD 8, PD-1, granzyme B, perforin, and EmG3) were conjugated with Cyanine3 (red). Additionally, each tissue section was stained with DAPI. The sections were examined under a fluorescence microscope; the dyes were excited at their respective wavelengths. Images were captured for each spectral range, and the three resulting images were overlaid.

Furthermore, we analyzed lymph nodes from patients infected with *E. granulosus* (IgG4: *n* = 8; IgE: *n* = 5), *E. multilocularis* (IgG4: *n* = 11; IgE: *n* = 13;), and non-infected individuals (IgG4: *n* = 15; IgE: *n* = 8). Tissue sections were stained immunohistochemically for IgG4, IgE, and EmG3. The infected lymph node sections were classified into three groups based on the intensity of EmG3 staining:

- “unaffected” indicated no EmG3 positivity;
- “moderate positivity” indicated EmG3 positivity in < 60% of germinal centers;
- “strong positivity” indicated EmG3 positivity in > 60% of germinal centers.

Subsequently, the number of IgG4- and IgE-positive cells was quantified in three high-power fields (400 × magnification) per section. The number of IgG4- and IgE-positive cells within the EmG3-defined groups was statistically compared to that of non-infected lymph nodes using a two-tailed *t*-test for heteroscedastic samples.

The same calculations and measurements were carried out on liver lesions of *E*. *granulosus* (*n *= 10) and *E*. *multilocularis* (*n *= 15) and were compared to liver sections of non-infected individuals. Patient characteristics are shown in Table 1.

**Table 1:** Patient data of stained liver lesions

	CE	AE	Control	Overall
Patients (*n*)	10	15	4	29
Male	6	6	2	14
Female	4	9	2	15
Male, mean age	40.8	53.5	49.0	47.4
Female, mean age	54.0	35.8	49.5	40.9
Overall, mean age	46.1	43	49.3	44.0

## Results

**Fig. 1:**
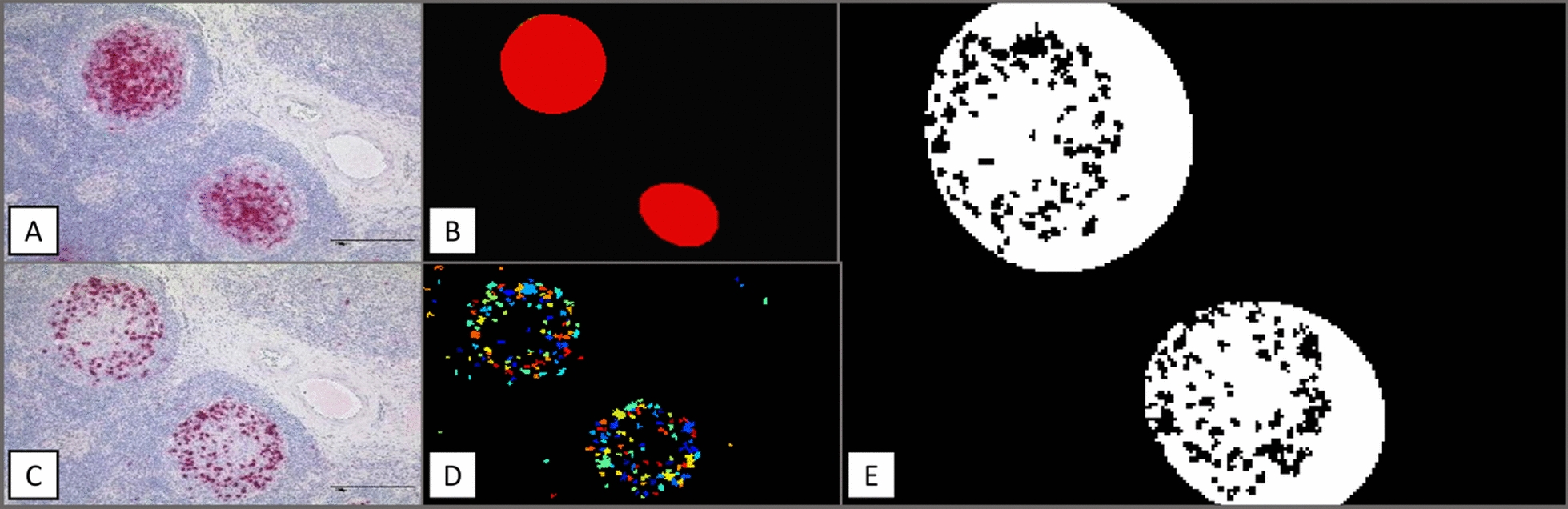
Processing of IHC staining using CellProfiler^™^ software. **A** EmG3-IHC of a lymph node from an *Echinococcus granulosus*-infected patient showing two EmG3-positive germinal centers. **B** Digital extraction of EmG3-positive germinal center areas highlighted in red. **C** IHC staining of the same lymph node using a CD57 antibody. **D** Identification and color-coded marking of all CD57-positive cells within the CD57-stained section. **E** Detection of CD57-positive cells located specifically within EmG3-positive germinal centers by digitally superimposing the segmented EmG3 signals from (**B**) with the CD57-positive cells from (**D**). Enlarged EmG3-positive germinal center areas (white circles) contain the CD57-positive cells (black dots) quantified in the final analysis

Software-based analysis of immunohistochemical stains from three lymph nodes of patients with CE identified 100 EmG3-positive and 16 EmG3-negative germinal centers. In three lymph nodes associated with AE, a total of 105 EmG3-positive and 30 EmG3-negative germinal centers were evaluated. This analysis revealed a significant increase in CD57-positive cells in EmG3-positive compared with EmG3-negative germinal centers, as well as a clear association between EmG3-positive particles and CD57-positive cells within the germinal centers of affected human lymph nodes (Fig. 1).

**Fig. 2:**
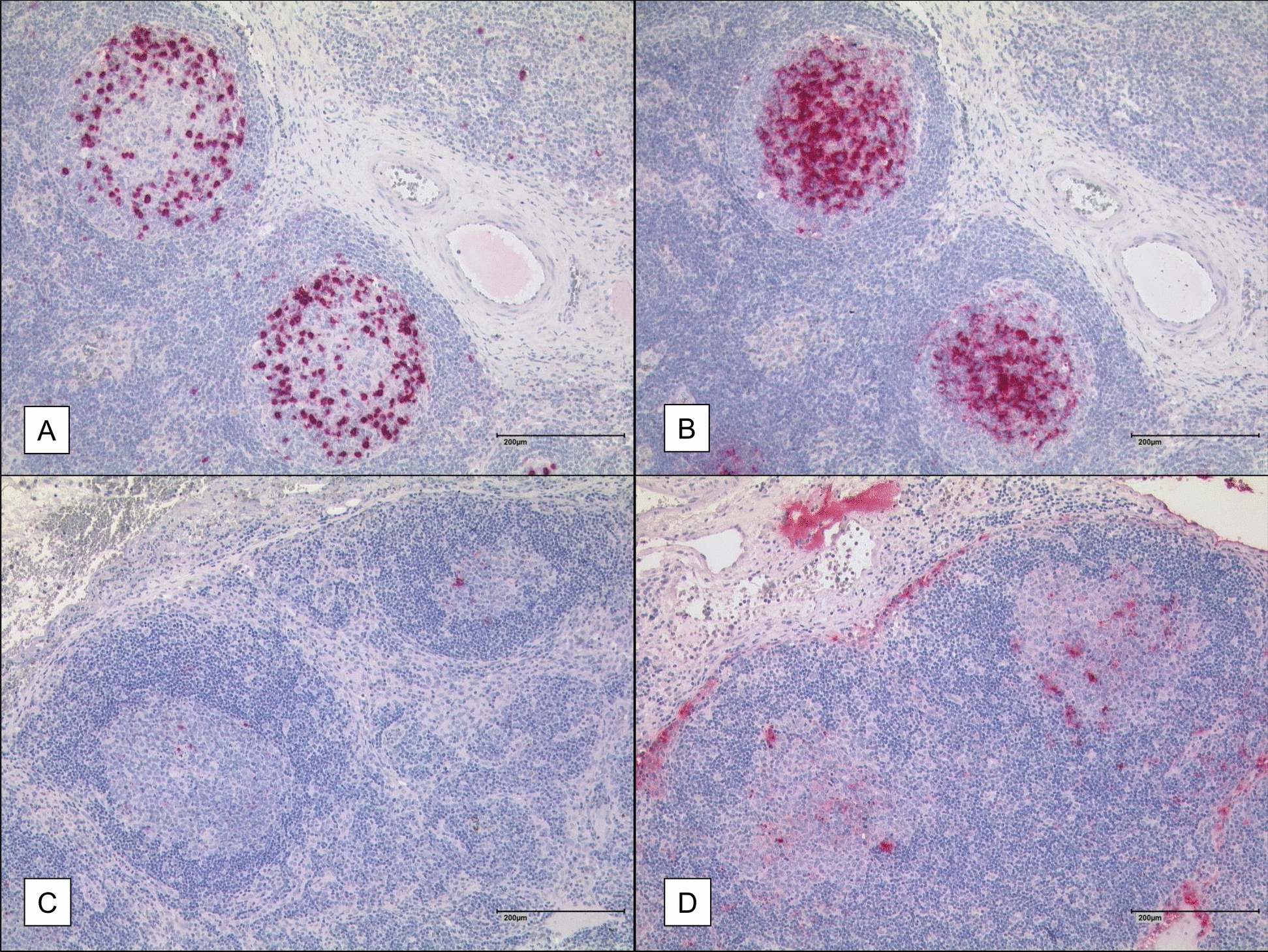
IHC of lymph nodes from *Echinococcus granulosus*-infected patients. **A** IHC staining using a CD57 antibody. **B** Immunohistochemical staining with the mAb EmG3 of the corresponding area shown in Fig. 2A, displaying strong EmG3 intensity associated with a high number of CD57-positive cells. **C** Overview of a lymph node negative for spegs with a few CD57-positive cells. **D** EmG3-IHC of the corresponding area shown in Fig. 2C, exhibiting low EmG3 intensity consistent with the reduced number of CD57-positive cells

**Fig. 3:**
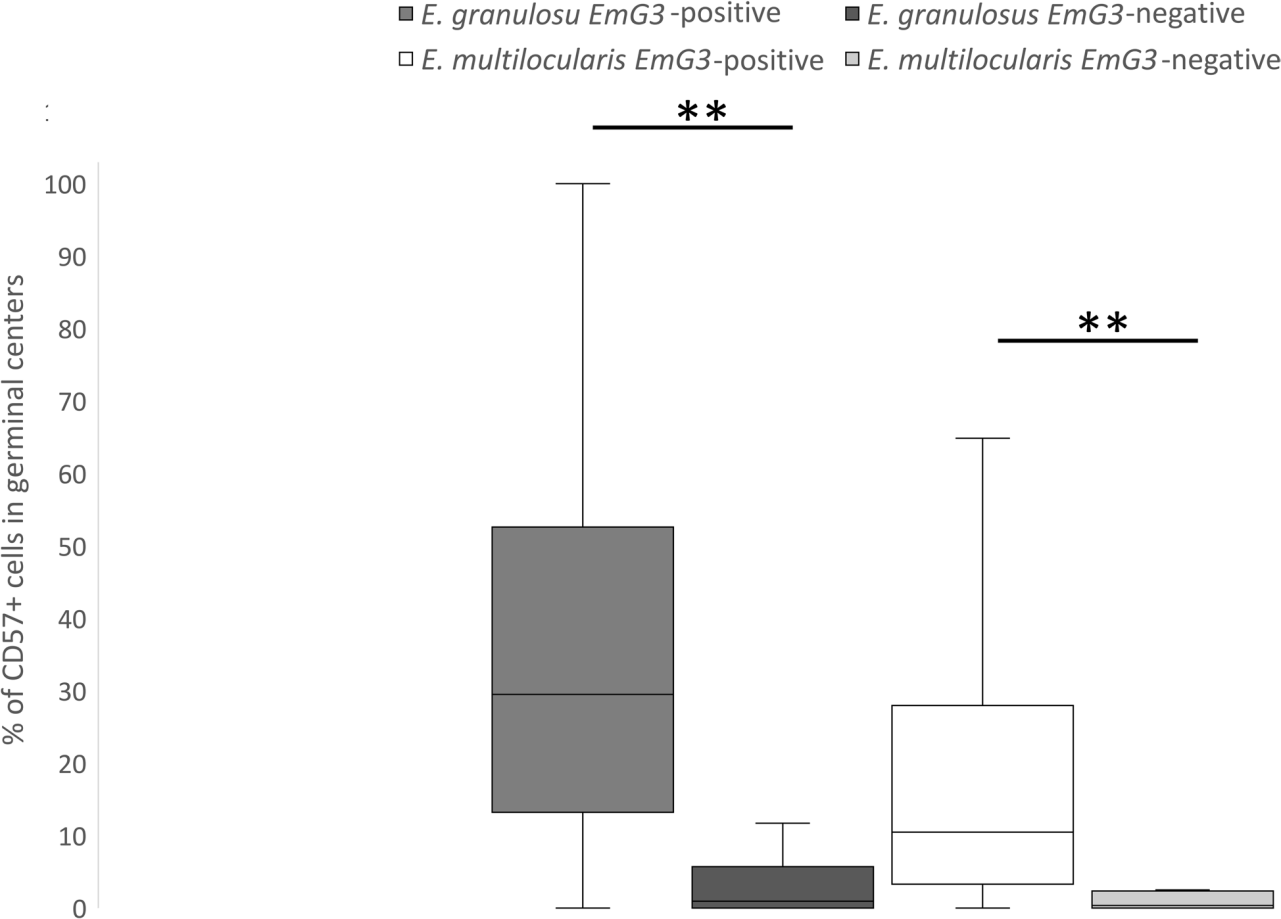
Range of the proportion of CD57-positive cells in EmG3-positive and EmG3-negative lymph nodes of patients infected with *Echinococcus granulosus* and *E. multilocularis*. Boxplots illustrate the number of CD57-positive cells in EmG3-positive and EmG3-negative germinal centers. The first two boxplots (dark gray = EmG3-positive, medium gray = EmG3-negative) represent lymph nodes from *E. granulosus*-infected patients, showing significantly fewer CD57-positive cells in EmG3-positive germinal centers. The third and fourth boxplots (white = EmG3 positive, light gray = EmG3 negative) display the corresponding comparison in lymph nodes from *E. multilocularis*-infected patients. Significance of differences are depicted as *p < 0.05 and **p < 0.01

EmG3-positive germinal centers contain a significantly higher CD57 +/total cells ratio than EmG3-negative lymph follicles, as shown in Figs. 2 and 3. Additionally, IHC staining of whole lymph nodes is shown in Fig. S1 in the supplementary material. The mean ratio of CD57-positive cells in lymph nodes of *E. granulosus*-infected patients was 33.8% in EmG3-positive follicles versus 3.6% in EmG3-negative follicles (t-test; t_(114)_ = 10.09; *p* < 0.0001). In lymph nodes of *E. multilocularis*-infected patients, the mean ratio of CD57 cell in EmG3-positive follicles was 19.0% versus 3.8% in EmG3-negative follicles (t-test; t_(133)_ = 5.77;* p* < 0.0001).

**Fig. 4:**
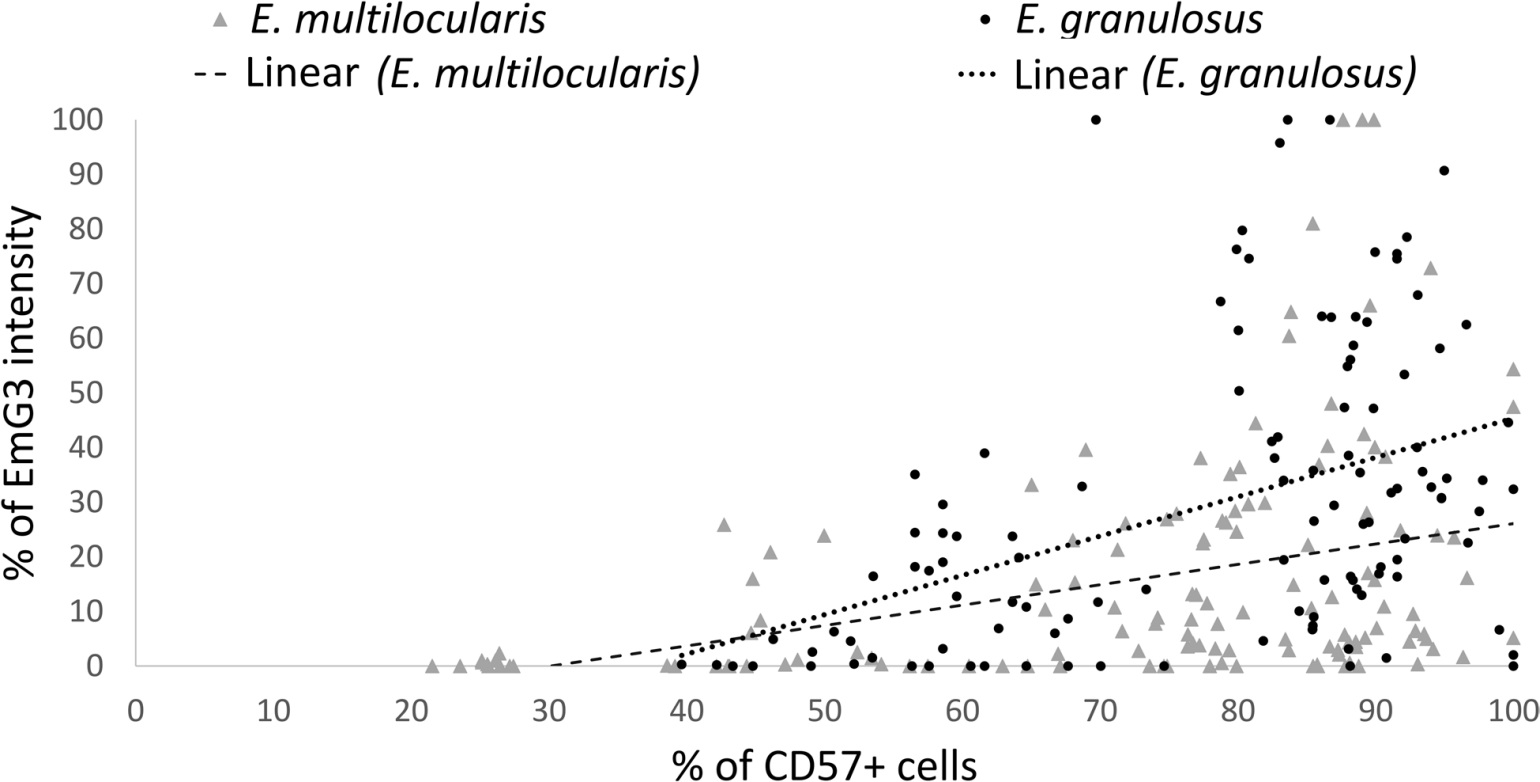
Correlation of EmG3 and CD57 staining. Dot plot showing the proportion of CD57-positive cells within lymph follicles, as a function of their intensity in mAb EmG3 staining, for probes of patients infected with *Echinococcus granulosus* (represented by triangles) or *E. multilocularis* (represented by dots). The intensity in EmG3 staining and the proportion of CD57-positive cells were normalized relative to the highest measurement per lymph node. An increase of CD57-positive cells dependent on EmG3 intensiity is shown by the regression line for *E. multilocularis* (dashed) and *E. granulosus* (dotted)

We found a moderate positive correlation between EmG3 intensity and number of CD57-positive cells for both species. *Echinococcus granulosus* (Spearman’s correlation coefficient, rs = 0.54; *p* < 0.0001) and *E. multilocularis* (Spearman’s correlation coefficient; rs = 0.59; *p* < 0.0001) (Fig. 4).

**Fig. 5:**
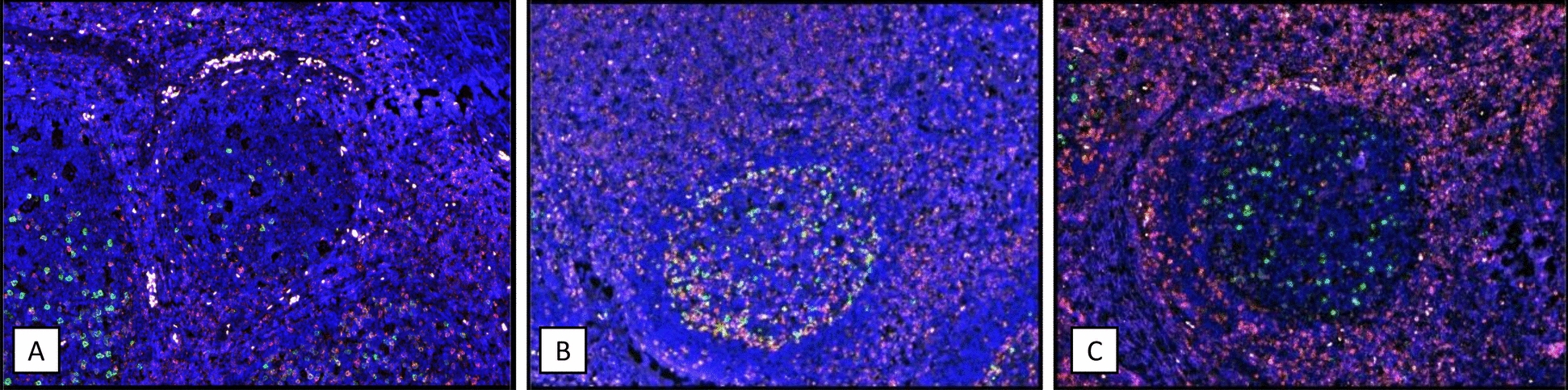
Double fluorescence histology using CD57 (green) and CD2 (red) antibodies. **A** Germinal center of a tonsil from a non-infected individual shows fewer CD57- and CD2-positive cells than a **B** germinal center of a lymph node affected by spegs or **C** a germinal center of a lymph node affected by spems

**Fig. 6:**
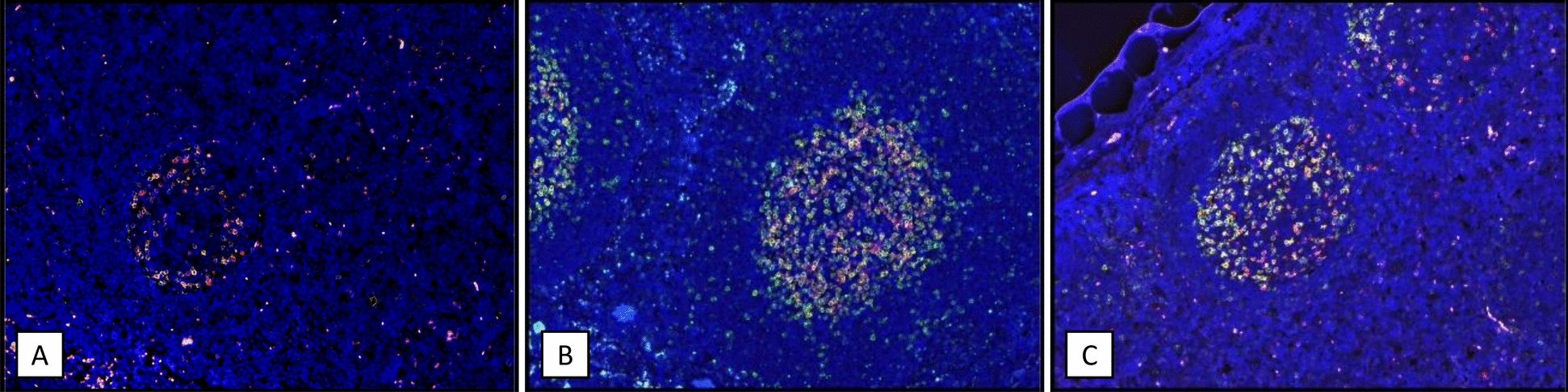
Double fluorescence histology using CD57 (green) and PD-1 (red) antibodies. **A** Germinal center of a tonsil from a non-infected individual shows fewer CD57- and PD-1-positive cells than a **B** germinal center of a lymph node affected by spegs and **C** a germinal center of a lymph node affected by spems

Immunofluorescence analysis of CD3 and CD57 of four lymph nodes (spegs or spems positive, *n *= 2 each) demonstrates a co-localization with a high expression of both markers on approximately 70% of CD57-positive cells. The spegs- and spems-positive germinal centers were identified by overlaying the immune-stained lymph nodes of serial paraffin sections. These CD57-/CD3-positive cells (Fig. S2) also expressed CD2, PD-1, and CD4. Germinal centers positive for spems or spegs contained a higher density of cells expressing these markers than germinal centers from tonsils of unaffected individuals (Figs. 5 and 6, Fig. S3). The remaining 30% of CD57-positive cells showed no or dim expression of CD8 (Fig. S4), perforin, and T cell intracellular antigen 1 (TIA1). No significant presence of perforin-, TIA1-, or granzyme B-positive cells was detected in any germinal center of any analyzed lymph node.

Further immunofluorescence analysis revealed elevated concentrations of both EmG3 and CD57 within the germinal centers of affected lymph nodes. EmG3 staining exhibited a diffuse extracellular distribution, whereas CD57 was primarily localized to the cell membrane. Notably, no increased concentration of EmG3 was observed on the surface of CD57-positive cells.

**Fig. 7:**
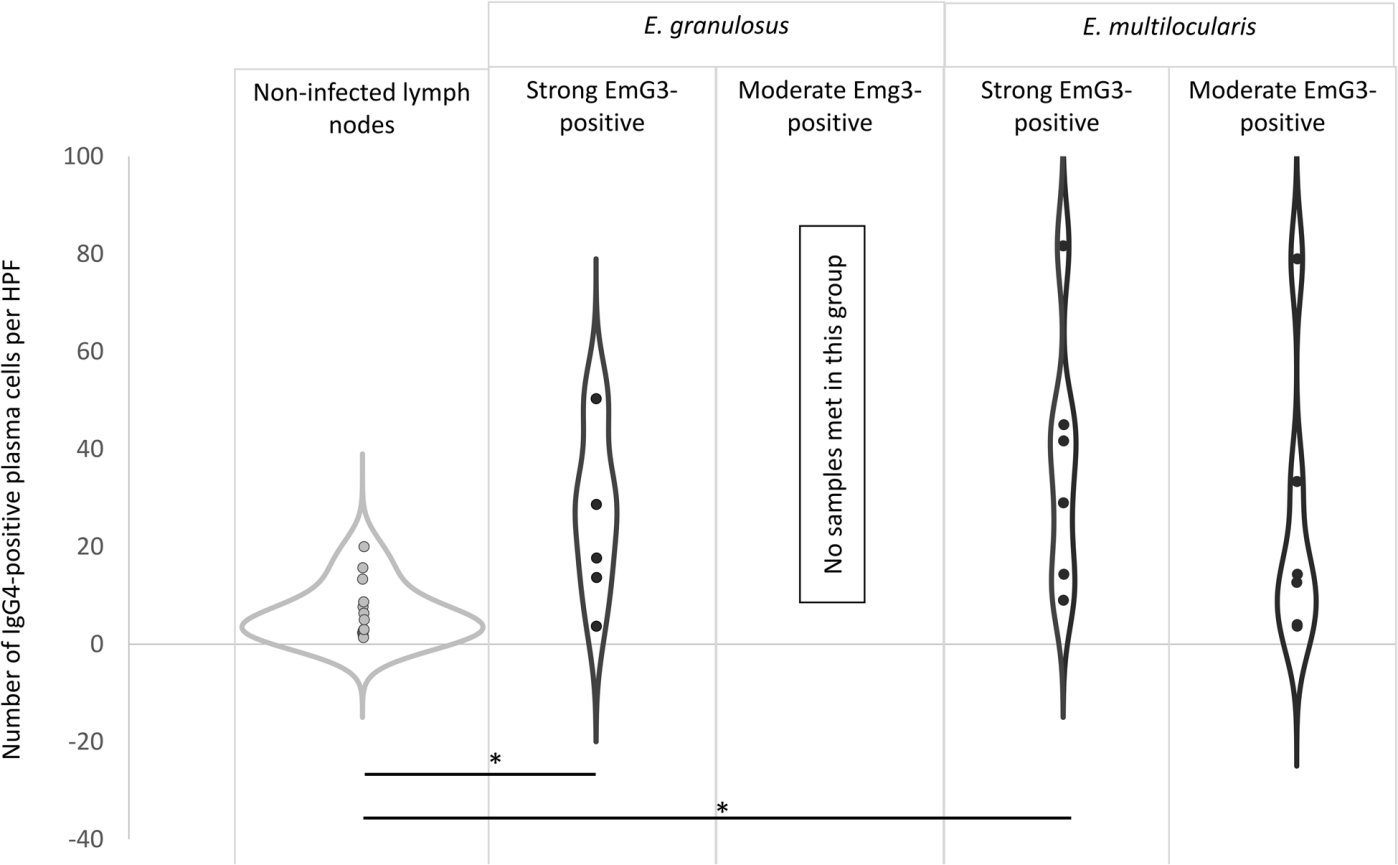
IgG4-positive plasma cells in lymph nodes of *Echinococcus granulosus* and *E. multilocularis* compared to non-infected individuals. Violin plots show the number of IgG4-positive cells per HPF (Y-axis) in non-affected lymph nodes, spegs-positive lymph nodes, and spems-positive lymph nodes. The non-affected group displays a markedly narrower distribution, whereas spegs- and spems-positive lymph nodes show a broader spread of IgG4-positive cell counts. Asterisks (*) indicate statistically significant differences compared to the respective non-affected control group ( *p* < 0.05). Since all *E. granulosus* lymph node sections showed > 60% EmG3-positive germinal center, no samples were found in the moderate EmG3 positivity group. *HPF* high-power field (400 × magnification)

The analysis of IgG4- and IgE-expressing plasma cells in lymph nodes affected by echinococcosis revealed a significant difference for IgG4 compared to non-affected lymph nodes: Lymph nodes with strong EmG3-positivity from patients infected with *E. granulosus* showed a significant increase in IgG4-positive plasma cells, with a mean of 22.8 (± 16.42) cells per high-power field compared to a mean of 6.3 (± 5.77) cells in non-affected lymph nodes (*t*-test, t_(21)_ = − 2.75; *p* = 0.007). In the group of lymph nodes from *E. multilocularis*-infected patients with high EmG3-intensity, the increase to a mean of 41.3 (± 28.09) IgG4-positive cells was statistically significant compared to non-affected lymph nodes (t-test, t_(18)_ = − 2.91; *p* = 0.007; *p* = 0.042). In lymph nodes from *E. multilocularis*-infected patients with moderate EmG3 positivity, an increase in IgG4-positive cells was also observed, with a mean of 24.5 (± 28.80) cells per high-power field. However, this increase did not reach statistical significance (Fig. 7).

**Fig. 8:**
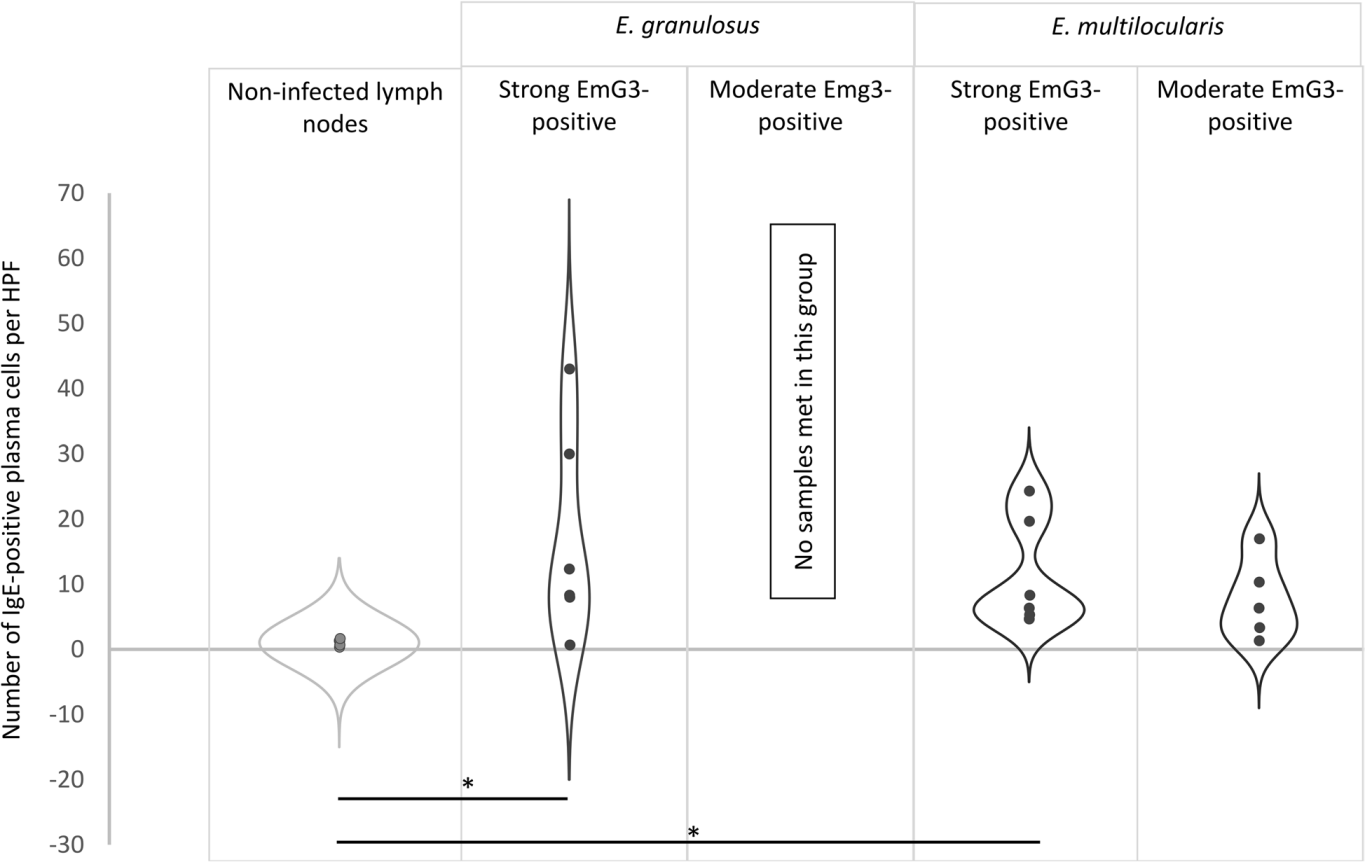
IgE-positive plasma cells in lymph nodes of *Echinococcus granulosus* and* E. multilocularis* compared to non-infected individuals. Violin plots show the number of IgGE-positive cells per HPF (Y-axis) in non-affected lymph nodes, spegs-positive lymph nodes, and spems-positive lymph nodes. The non-affected group displays a markedly narrower distribution, whereas spegs- and spems-positive lymph nodes show a broader spread of IgGE-positive cell counts. Asterisks (*) indicate statistically significant differences compared to the respective non-affected control group (*p < 0.05). Since all *E. granulosus* lymph node sections showed a > 60% portion of EmG3-positive germinal center, no samples were found in the group of moderate EmG3 positivity.* HPF* high-power field (400 × magnification)

Analysis of IgE expression revealed the following results: Lymph nodes with strong EmG3 positivity from patients infected with *E. granulosus* showed a mean of 20.3 (± 15.55) IgE-positive cells per high-power field compared to 1.0 (± 0.52) cells in non-affected lymph nodes (*t*-test, t_(11)_ = − 2.78; *p* = 0.049). In the group of lymph nodes from *E. multilocularis*-infected patients, a mean of 11.4 (± 8.40) IgE-positive cells per high-power field was observed, which was statistically significant compared to non-affected lymph nodes (*t*-test, t_(12)_ = − 3.05; *p* = 0.028). In lymph nodes with moderate EmG3 positivity from *E. multilocularis*-infected patients, a mean of 7.6 (± 6.22) IgE-positive cells per high-power field was found; this difference was not statistically significant compared to non-affected lymph nodes (*t*-test, t_(11)_ = − 2.41; *p* = 0.073). No lymph nodes with moderate EmG3 positivity were present in the group of *E. granulosus*-infected patients (Fig. 8).

The analysis of IgG4- and IgE-stained liver lesions from *E. granulosus*- and *E. multilocularis*-infected patients revealed significant differences in the number of positive plasma cells compared to non-infected liver tissue.

The number of IgG4-positive plasma cells was significantly elevated in *E. granulosus*-infected sections, with an average of 39.1 (± 24.9) cells (*t*-test, t_(12)_ = − 4.59; *p* = 0.0017), and in *E. multilocularis*-infected sections, with an average of 28.1 (± 15.8) cells (*t*-test, t_(18)_ = − 6.64; *p* < 0.0001), compared to an average of approximately 1 (± 0.6) IgG4-positive plasma cell per section in non-infected liver tissue.

**Fig. 9:**
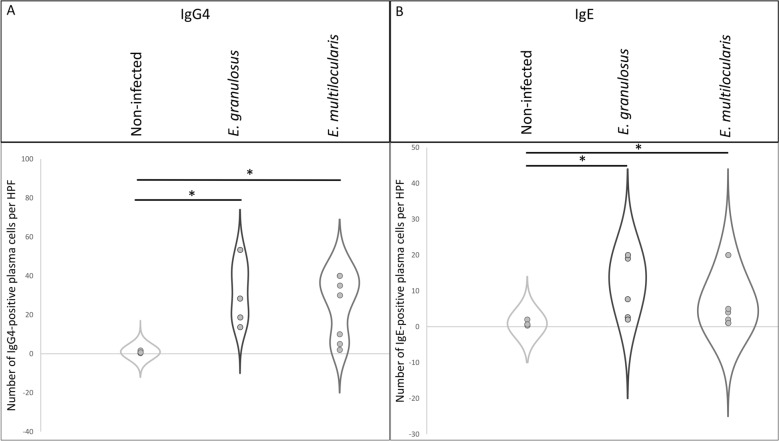
IgG4- and IgE-expression liver lesions of *Echinococcus granulosus* and *E. multilocularis* compared to non-infected individuals. **A** Violin plots showing the number of IgG4-positive cells per HPF (Y-axis) in non-affected liver sections. *Echinococcus granulosus* and *E. multilocularis* caused liver lesions. The non-affected group displays a markedly narrower distribution, whereas *E. granulosus*- and *E. multilocularis*-infected liver sections show a broader spread of IgG4-positive cell counts. **B** Violin plots showing the number of IgE-positive cells per HPF (Y-axis) in the same groups, again with lower variability in non-affected lymph nodes. Asterisks (*) indicate statistically significant differences compared to the respective non-affected control group (*p < 0.05). HPF: high-power field (400 × magnification)

A similar pattern was observed for IgE-positive plasma cells. Liver sections infected with *E. granulosus* and *E. multilocularis* showed average counts of 12.8 (± 7.11) and 6.6 (± 6.62) IgE-positive plasma cells, respectively, whereas non-infected liver tissue displayed an average of only 0.9 cells (Fig. 9).

Moreover, the number of IgE-positive plasma cells was significantly higher in *E. granulosus*-infected than in *E. multilocularis*-infected tissue (t-test, t_(13)_ = − 5.24; *p* = 0.015). No significant difference in IgG4-positive plasma cells was observed between the two groups.

## Discussion

Our computational analysis revealed an increased density of CD57-positive cells in the germinal centers of lymph nodes containing spems and spegs, as assessed by double immunofluorescence. These cells were further characterized as CD2-, CD3-, and CD4-positive T cells expressing PD-1, consistent with a Tfh cell phenotype. We acknowledge that our histological and topographical definition did not include additional molecular Tfh markers such as CXCR5, BCL6, ICOS, or IL-21. However, the identification of CD4⁺ T cells with high PD-1 expression and CD57 positivity localized within germinal centers provides strong histological evidence for bona fide T-follicular helper cells. To our knowledge, no alternative CD4⁺ T-cell population displays this specific phenotypic and spatial profile within germinal centers. CD57 expression has previously been described as a feature of a mature or highly differentiated subset of Tfh cells, further supporting our interpretation [23]. These findings align with the essential role of Tfh cells in germinal centers, where they support B cell maturation and promote the production of high-affinity antibodies [24, 25].

We have previously shown that spems and spegs interact with CD23-positive dendritic cells [8]. In the context of *Echinococcus* infections, antigen-presenting cells, such as dendritic cells, can internalize parasite-derived antigens, including spegs and spems [6, 26, 27].

Alterations in germinal center architecture during *Echinococcus* infections have been demonstrated previously, showing that spems and spegs accumulate in the germinal centers of infected individuals [8]. Importantly, this accumulation does not appear to be merely passive, as lymph nodes containing these particles exhibit a significant increase in size [8]. Germinal centers are highly dependent on Tfh cells for their formation and maintenance [28]. Our study further establishes an association between spems and spegs and CD57-positive Tfh cells. Parasite-associated modulation of this key cell population may substantially influence the host immune response. These Tfh cells retain limited cytotoxic capacity; instead, their primary function is to shape the plasma cell response by supporting B-cell maturation, differentiation, and affinity maturation within germinal centers. [29–31]

One mechanism by which Tfh cells shape immune responses is by guiding B cells to undergo class switch recombination toward specific immunoglobulin isotypes, including IgG4 and IgE [32–34]. The critical role of Tfh cells in B-cell responses has been demonstrated in vivo, where they are required for antibody class switching, even prior to germinal center formation [25]. In the context of echinococcosis, this function is particularly relevant, as IgG4 and IgE play important roles in immune responses to parasitic infections [20, 35].

For *Echinococcus*-derived particles such as spegs and spems, immunomodulatory effects have been reported previously [36]. Studies in murine models have demonstrated that these particles can impair dendritic cell and macrophage function [37, 38]. Various helminths are known to modulate the Th1/Th2 immune balance [39]. CD57-positive cells may contribute to immune modulation rather than solely immune activation or inhibition, as these cells express a Th1-associated cytokine profile while retaining IL-12 expression [14]. In the presence of IL-12, a Th1 response is characterized by cytokines such as IFN-γ, IL-6, IL-12, and TNF-α [38, 39]. In *Echinococcus* infections, a Th1 response has been associated with protective immunity, whereas a Th2 response (IL-4, IL-5) correlates with disease progression [39–41].

At the same time, PD-1 expression on these cells suggests additional inhibitory effects on immune activity, potentially contributing to parasite immune evasion strategies [15].

This hypothesis is supported by observations in other helminth infections, in which expansion of CD57-positive T cells in germinal centers or peripheral blood has been linked to pathogen persistence [15, 18, 42, 43]. In alveolar echinococcosis, CD57 expression has been observed in hepatic tissue, further highlighting its potential role in modulating immune responses during chronic infection [44].

In addition to a significant increase in IgG4- and IgE-positive plasma cells within involved lymph nodes, we observed elevated numbers of these cells in the perilesional areas of corresponding liver lesions. These data therefore suggest an association between spem- and speg-positive lymph nodes and the presence of specific plasma cell subsets accumulating around hepatic lesions, potentially within a cytokine milieu surrounding the primary lesion [35, 45, 46].

In contrast to IgG4, which exhibits predominantly immunosuppressive properties, IgE is associated with a strong proinflammatory immune response [47]. Nevertheless, both antibody classes are induced by a similar cytokine environment, predominantly IL-4 and IL-13, and may occur in parallel during chronic parasitic infections [47, 48].

Taken together, *E. multilocularis* and *E. granulosus* appear to be associated with immunomodulatory processes that coincide with a host immune state intermediate between activation and suppression. Immune responses potentially detrimental to the parasite, such as eosinophil recruitment, have been reported to be reduced. Accordingly, hepatic lesions in alveolar echinococcosis show no significant eosinophilic infiltration despite elevated IgE concentrations [49].

## Conclusions

Our findings indicate that echinococcosis involves a complex interaction with the human immune system, involving both immunostimulatory and immunomodulatory processes. We observed an increased presence of CD57-positive Tfh cells in lymph nodes containing spems and spegs, highlighting an association between this cell population and immune responses to *E. multilocularis* and *E. granulosus*. A state of chronic, low-level inflammation, reflected by the mechanisms described here, may be relevant for immune evasion and could potentially contribute to parasite persistence within the host.

Accordingly, our data suggest an association between parasite presence and immune regulatory processes that coincide with attenuation of proinflammatory responses and may support chronic host-parasite coexistence. Spegs and spems were identified as being associated with these observations; however, additional parasite-derived metabolites not captured by our analysis may also contribute. Further functional and mechanistic studies, including multicenter cohorts, will be required to clarify the causal relationships underlying these findings.

## Supplementary Information

Supplementary Material 1. Fig. S1: IHC of lymph nodes from *Echinococcus granulosus*-infected patients. **A** IHC staining using a CD57 antibody. In the insert, an enlargement of the labeled area, including a germinal center, is shown. **B** Immunohistochemical staining with the mAb EmG3- of the corresponding area depicted in **A**. In the insert, an enlargement of the labeled area is displayed. **C** Overview of a lymph node negative for spegs with a few CD57-positive cells observed in the IHC using a CD57 antibody. **D** EmG3-IHC of the corresponding lymph node depicted in **C**

Supplementary Material 2. Fig. S2: Double fluorescent IHC using CD57 (green) and CD3 (red) antibodies. **A** Germinal center of a tonsil from an uninfected individual. **B** Germinal center of a lymph node affected by spegs. **C** Germinal center of a lymph node affected by spems.

Supplementary Material 3. Fig. S3: Double fluorescent IHC using CD57 (green) and CD4 (red) antibodies. **A** Germinal center of a tonsil from an uninfected individual. **B** Germinal center of a lymph node affected by spegs. **C** Germinal center of a lymph node affected by spems.

Supplementary Material 4. Fig. S4: Double fluorescent IHC using CD57 (green) and CD8 (red) antibodies. **A** Germinal center of a tonsil from an uninfected individual. **B** Germinal center of a lymph node affected by spegs. **C** Germinal center of a lymph node affected by spems.

Supplementary Material 5.

Supplementary Material 6.

Supplementary Material 7.

## Data Availability

Data supporting the main conclusions of this study are included in the manuscript.

## Abbreviations

AE: Alveolar echinococcosis
CE: Cystic echinococcosis
CD: Cluster of differentiation
IHC: Immunohistochemistry
mAb: Monoclonal antibody
NK: Natural killer cells
PAS: Periodic acid-Schiff staining
PD-1: Programmed cell death protein 1
spegs: Small particles of *Echinococcus granulosus*
spems: Small particles of *Echinococcus multilocularis*
TIA1: T cell intracellular antigen 1
Tfh: T follicular helper cells
LL: Laminated layer
CE: Cystic echinococcosis
AE: Alveolar echinococcosis
TCRs: T cell receptors
APCs: Antigen presenting cells
DCs: Dendritic cells
HPF: High power field (400 × magnification)
