# Supplementary material for: Small particles of Echinococcus granulosus (spegs) and Echinococcus multilocularis (spems) promote follicular T helper cell expansion and are associated with IgE and IgG4 class switching in human lymph nodes
Source: Parasit Vectors. 2026 Mar 18;19:151. doi: 10.1186/s13071-026-07321-4 (PMC13064062; doi:10.1186/s13071-026-07321-4)
Supplement: Supplementary file 5 — Supplementary Material 5. [file 13071_2026_7321_MOESM5_ESM.docx]

**Table S1:** Provider and dilution of the antibodies used in immunohistochemistry and double immunofluorescent staining.

| Name | Specification/provider | Dilution |
| --- | --- | --- |
| EmG3 | Peter Deplazes. Zürich. Switzerland [7.11.22] | 1:2000 |
| CD3 | Agilent DAKO; Number: M7254 | 1:100 |
| CD57 | Zytomed Systems; Number: ZYT-CM007B | 1:50 |
| TIA-1 | Zytomed Systems; Number: 120-0191 | 1:50 |
| CD2 | Agilent DAKO; Number: GA651 | Ready-to-use |
| CD4 | Agilent DAKO; Number: M731 | 1:50 |
| CD8 | Agilent DAKO; Number: M7103 | 1:150 |
| Granzyme B | Agilent DAKO; Number: M7235 | 1:25 |
| Perforin | Menarini; Number: NCL-Perforin | Ready-to-use |
| PD-1 | Dianova; Number: DLN-14587 | Ready-to-use |
| IgG4 | Zytomed Systems; Number: MSK084-05 | 1:50 |
| IgE | thermos scientific; Number: RB-1435-A | 1:200 |
