# Supplementary material for: Small particles of Echinococcus granulosus (spegs) and Echinococcus multilocularis (spems) promote follicular T helper cell expansion and are associated with IgE and IgG4 class switching in human lymph nodes
Source: Parasit Vectors. 2026 Mar 18;19:151. doi: 10.1186/s13071-026-07321-4 (PMC13064062; doi:10.1186/s13071-026-07321-4)
Supplement: Supplementary file 6 — Supplementary Material 6. [file 13071_2026_7321_MOESM6_ESM.docx]

**Table S2: Patients' characteristics and localization of the primary lesion of lymph nodes used in the computer analysis.**

|  | AE | CE | Overall |
| --- | --- | --- | --- |
| Patients (n) | 3 | 3 | 6 |
| Male | 1 | 2 | 3 |
| Female | 2 | 1 | 3 |
| Male; mean age (range)/mean | (28)/28 | (18-35)26.5 | (18-35)/27 |
| Female; mean age (range)/mean | (17-63)/40 | (40)/40 | (17-63)/40 |
| Overall; mean age (range)/mean | (17-63)/36 | (18-40)/35 | (17-63)/33.5 |
| Primary lesion |  |  |  |
| -liver | 3 | 1 | 4 |
| -lung | 0 | 2 | 2 |
