# Supplementary material for: Small particles of Echinococcus granulosus (spegs) and Echinococcus multilocularis (spems) promote follicular T helper cell expansion and are associated with IgE and IgG4 class switching in human lymph nodes
Source: Parasit Vectors. 2026 Mar 18;19:151. doi: 10.1186/s13071-026-07321-4 (PMC13064062; doi:10.1186/s13071-026-07321-4)
Supplement: Supplementary file 7 — Supplementary Material 7. [file 13071_2026_7321_MOESM7_ESM.docx]

**Table S3: Patients' characteristics and localization of the primary lesion of lymph nodes used in the fluorescent staining.**

|  | AE | CE | Overall |
| --- | --- | --- | --- |
| Patients (n) | 2 | 2 | 4 |
| Male | 0 | 1 | 2 |
| Female | 2 | 1 | 2 |
| Male; mean age (range)/mean | (-)- | (26)/26 | (26)/26 |
| Female; mean age (range)/mean | (17-49)/33 | (64)/64 | (17-64)/43.3 |
| Overall; mean age (range)/mean | (17-64)/33 | (26-64)/45 | (17-64)/39 |
| Primary lesion |  |  |  |
| -liver | 2 | 2 | 4 |
| -lung | 0 | 0 | 0 |
